# Supplementary material for: A new Mentor Evaluation Tool: Evidence of validity
Source: PLoS One. 2020 Jun 16;15(6):e0234345. doi: 10.1371/journal.pone.0234345 (PMC7297334; doi:10.1371/journal.pone.0234345)
Supplement: S1 Appendix — (PDF) [file pone.0234345.s001.pdf]

S1 Appendix 1 Mentor Evaluation Tool:

Q1 This survey allows mentees to evaluate their mentors with a confidential, standardized, quantitative instrument. It takes about 5 minutes to complete.

Q2 First name of your mentor.
Q3 Last name of your mentor.Q4 Your mentor's school.

- School of Dentistry
- School of Medicine
- School of Nursing
- School of Pharmacy

Q5 Your academic position.

- Faculty member
- Resident, fellow or postdoc
- Student

If resident, fellow, postdoc or student was selected Qualtrics branching automatically skipped to question 9.

Q6 If you are a faculty member, please select your rank:

- Instructor
- Assistant Professor
- Associate Professor
- Professor

Q7 Your faculty series:

- Adjunct
- Clinical X
- Health Sciences Clinical
- In Residence
- Ladder Rank
- Not Sure

Q8 Your faculty step (Three step levels for Assistant Professor, Three step levels for Associate Professor and up to 9 step levels for Professor):

- ❍ 1
- ❍ 2
- ❍ 3
- ❍ 4
- ❍ 5
- ❍ 6
- ❍ 7
- ❍ 8
- ❍ 9
- ❍ Above scale
- ❍ Not Sure

Q9 How many years has this person been your mentor?

- ❑ Less than one year
- ❑ 1 – 2 years
- ❑ 2 – 3 years
- ❑ 3 years or more

Q10 Was this mentor assigned to you or someone you found on your own?

- ❍ Assigned
- ❍ I found myself
- ❍ Other (please explain): ____________________

Q11 What role does this mentor provide for you? (select all that apply)

- ❑ Research/Scholarly Mentor Provides overall research and/or scholarly guidance.
- ❑ Project Mentor Supervises a defined, time-limited project, e.g. data collection, data analysis, manuscript preparation, grant preparation, curriculum or course development.
- ❑ Career Mentor Provides overall career guidance and support mentoring.
- ❑ Co-Mentor or Clinical Mentor Provides specialized, content area, methodological or clinical expertise as part of a mentoring research or clinical team.

Q12 Please indicate how strongly you agree or disagree with the statements below.

	Strongly Disagree (-3)	Dis-agree (-2)	Slightly Disagree (-1)	Neither Disagree or Agree (0)	Slightly Agree (1)	Agree (2)	Strongly Agree (3)	NA (-99)
My mentor is accessible. (1)	❍	❍	❍	❍	❍	❍	❍	❍
My mentor is an active listener. (2)	❍	❍	❍	❍	❍	❍	❍	❍
My mentor demonstrates professional expertise. (3)	❍	❍	❍	❍	❍	❍	❍	❍
My mentor encourages me to establish an independent career. (4)	❍	❍	❍	❍	❍	❍	❍	❍
My mentor provides useful critiques of my work. (5)	❍	❍	❍	❍	❍	❍	❍	❍
My mentor motivates me to improve my work. (6)	❍	❍	❍	❍	❍	❍	❍	❍
My mentor is helpful in providing direction and guidance on professional issues. (7)	❍	❍	❍	❍	❍	❍	❍	❍
My mentor acknowledges my contributions appropriately. (8)	❍	❍	❍	❍	❍	❍	❍	❍

My mentor takes a sincere interest in my career. (9)	❍	❍	❍	❍	❍	❍	❍	❍
My mentor helps me to formulate clear goals. (10)	❍	❍	❍	❍	❍	❍	❍	❍
My mentor facilitates building my professional network. (11)	❍	❍	❍	❍	❍	❍	❍	❍
My mentor provides thoughtful advice on my scholarly work. (12)	❍	❍	❍	❍	❍	❍	❍	❍
My mentor is supportive of work-life balance. (13)	❍	❍	❍	❍	❍	❍	❍	❍
Overall, I'm satisfied with my mentor. (14)	❍	❍	❍	❍	❍	❍	❍	❍

Q13 To SAVE and SUBMIT your responses, please click the right arrow. If you want to exit without saving your responses, close this window without clicking the arrow.
