# Supplementary material for: A new Mentor Evaluation Tool: Evidence of validity
Source: PLoS One. 2020 Jun 16;15(6):e0234345. doi: 10.1371/journal.pone.0234345 (PMC7297334; doi:10.1371/journal.pone.0234345)
Supplement: S1 Dataset — (PDF) [file pone.0234345.s002.pdf]

MentorID	Mentor Group	Years Mentoring	Your academic position.	How many years has this person been your mentor?
2	LAMA	45	Student	3 years or more
2	LAMA	45	Faculty member	3 years or more
2	LAMA	45	Student	3 years or more
2	LAMA	45	Student	3 years or more
10	LAMA	30	Faculty member	3 years or more
10	LAMA	30	Faculty member	3 years or more
11	LAMA	18	Faculty member	3 years or more
11	LAMA	18	Faculty member	3 years or more
14	LAMA	37	Faculty member	3 years or more
14	LAMA	37	Faculty member	3 years or more
17	LAMA	30	Resident, fellow or postdoc	2 to 3 years
17	LAMA	30	Resident, fellow or postdoc	3 years or more
17	LAMA	30	Faculty member	3 years or more
17	LAMA	30	Faculty member	3 years or more
23	LAMA	.	Faculty member	3 years or more
30	LAMA	15	Faculty member	3 years or more
30	LAMA	15	Faculty member	3 years or more
30	LAMA	15	Faculty member	3 years or more
33	LAMA	40	Faculty member	3 years or more
33	LAMA	40	Faculty member	3 years or more
33	LAMA	40	Faculty member	3 years or more
35	LAMA	25	Faculty member	3 years or more
35	LAMA	25	Faculty member	1 to 2 years
36	LAMA	31	Faculty member	3 years or more
36	LAMA	31	Resident, fellow or postdoc	3 years or more
36	LAMA	31	Faculty member	3 years or more
38	LAMA	53	Faculty member	3 years or more
38	LAMA	53	Faculty member	3 years or more
38	LAMA	53	Faculty member	3 years or more
41	LAMA	27	Faculty member	3 years or more
41	LAMA	27	Resident, fellow or postdoc	2 to 3 years
41	LAMA	27	Resident, fellow or postdoc	2 to 3 years

41	LAMA	27	Faculty member	3 years or more
46	LAMA	30	Faculty member	3 years or more
46	LAMA	30	Resident, fellow or postdoc	3 years or more
46	LAMA	30	Student	1 to 2 years
46	LAMA	30	Faculty member	3 years or more
49	LAMA	37	Resident, fellow or postdoc	3 years or more
49	LAMA	37	Faculty member	3 years or more
49	LAMA	37	Student	3 years or more
56	LAMA	29	Student	3 years or more
56	LAMA	29	Faculty member	3 years or more
59	LAMA	40	Faculty member	3 years or more
1	MDP	9	Student	2 to 3 years
1	MDP	9	Faculty member	3 years or more
1	MDP	9	Faculty member	3 years or more
1	MDP	9	Faculty member	3 years or more
3	MDP	10	Faculty member	2 to 3 years
3	MDP	10	Resident, fellow or postdoc	3 years or more
3	MDP	10	Faculty member	3 years or more
4	MDP	.	Faculty member	3 years or more
7	MDP	.	Faculty member	3 years or more
8	MDP	8	Faculty member	2 to 3 years
9	MDP	15	Faculty member	1 to 2 years
9	MDP	15	Faculty member	2 to 3 years
9	MDP	15	Student	1 to 2 years
12	MDP	7	Faculty member	2 to 3 years
12	MDP	7	Faculty member	3 years or more
15	MDP	12	Student	Less than one year
15	MDP	12	Faculty member	3 years or more
15	MDP	12	Faculty member	3 years or more
16	MDP	4	Resident, fellow or postdoc	1 to 2 years
18	MDP	10	Faculty member	3 years or more
18	MDP	10	Faculty member	3 years or more
19	MDP	20	Resident, fellow or postdoc	3 years or more
22	MDP	8	Faculty member	3 years or more
22	MDP	8	Resident, fellow or postdoc	3 years or more
22	MDP	8	Faculty member	3 years or more
24	MDP	6	Resident, fellow or postdoc	2 to 3 years

24	MDP	6	Resident, fellow or postdoc	1 to 2 years
24	MDP	6	Resident, fellow or postdoc	2 to 3 years
24	MDP	6	Resident, fellow or postdoc	3 years or more
24	MDP	6	Faculty member	1 to 2 years
25	MDP	5	Faculty member	3 years or more
26	MDP	10	Resident, fellow or postdoc	2 to 3 years
27	MDP	.	Faculty member	3 years or more
28	MDP	12	Resident, fellow or postdoc	2 to 3 years
31	MDP	.	Faculty member	2 to 3 years
31	MDP	.	Faculty member	2 to 3 years
31	MDP	.	Faculty member	3 years or more
31	MDP	.	Faculty member	Less than one year
34	MDP	8	Faculty member	3 years or more
37	MDP	15	Resident, fellow or postdoc	3 years or more
37	MDP	15	Resident, fellow or postdoc	3 years or more
37	MDP	15	Faculty member	3 years or more
39	MDP	15	Faculty member	3 years or more
39	MDP	15	Faculty member	3 years or more
45	MDP	.	Resident, fellow or postdoc	Less than one year
45	MDP	.	Resident, fellow or postdoc	1 to 2 years
50	MDP	7	Resident, fellow or postdoc	1 to 2 years
50	MDP	7	Faculty member	2 to 3 years
51	MDP	15	Faculty member	3 years or more
51	MDP	15	Resident, fellow or postdoc	2 to 3 years
52	MDP	10	Student	1 to 2 years
52	MDP	10	Student	Less than one year
52	MDP	10	Faculty member	Less than one year
52	MDP	10	Student	2 to 3 years
54	MDP	12	Faculty member	3 years or more
55	MDP	12	Resident, fellow or postdoc	2 to 3 years
55	MDP	12	Resident, fellow or postdoc	1 to 2 years

55	MDP	12	Faculty member	3 years or more
56	MDP	29	Faculty member	3 years or more
60	MDP	12	Faculty member	2 to 3 years
60	MDP	12	Faculty member	3 years or more
61	MDP	7	Faculty member	3 years or more
61	MDP	7	Resident, fellow or postdoc	3 years or more
61	MDP	7	Faculty member	3 years or more
5	Non-LAMA/MDP	15	Faculty member	3 years or more
5	Non-LAMA/MDP	15	Student	3 years or more
6	Non-LAMA/MDP	6	Faculty member	2 to 3 years
6	Non-LAMA/MDP	6	Faculty member	2 to 3 years
6	Non-LAMA/MDP	6	Resident, fellow or postdoc	2 to 3 years
6	Non-LAMA/MDP	6	Faculty member	3 years or more
13	Non-LAMA/MDP	10	Faculty member	3 years or more
13	Non-LAMA/MDP	10	Faculty member	Less than one year
13	Non-LAMA/MDP	10	Faculty member	3 years or more
20	Non-LAMA/MDP	40	Faculty member	3 years or more
20	Non-LAMA/MDP	40	Faculty member	3 years or more
21	Non-LAMA/MDP	10	Resident, fellow or postdoc	3 years or more
21	Non-LAMA/MDP	10	Faculty member	3 years or more
29	Non-LAMA/MDP	21	Resident, fellow or postdoc	3 years or more
29	Non-LAMA/MDP	21	Resident, fellow or postdoc	3 years or more
32	Non-LAMA/MDP	20	Faculty member	3 years or more
32	Non-LAMA/MDP	20	Resident, fellow or postdoc	3 years or more
32	Non-LAMA/MDP	20	Faculty member	1 to 2 years
40	Non-LAMA/MDP	42	Faculty member	3 years or more
40	Non-LAMA/MDP	42	Student	2 to 3 years
40	Non-LAMA/MDP	42	Faculty member	3 years or more
40	Non-LAMA/MDP	42	Resident, fellow or postdoc	3 years or more
42	Non-LAMA/MDP	10	Faculty member	3 years or more
42	Non-LAMA/MDP	10	Faculty member	2 to 3 years
42	Non-LAMA/MDP	10	Faculty member	2 to 3 years
42	Non-LAMA/MDP	10	Faculty member	2 to 3 years
42	Non-LAMA/MDP	10	Faculty member	2 to 3 years
43	Non-LAMA/MDP	15	Faculty member	3 years or more
44	Non-LAMA/MDP	7	Faculty member	3 years or more
47	Non-LAMA/MDP	7	Faculty member	3 years or more
47	Non-LAMA/MDP	7	Faculty member	Less than one year
47	Non-LAMA/MDP	7	Faculty member	3 years or more
48	Non-LAMA/MDP	30	Faculty member	3 years or more
48	Non-LAMA/MDP	30	Faculty member	3 years or more
48	Non-LAMA/MDP	30	Faculty member	3 years or more
48	Non-LAMA/MDP	30	Resident, fellow or postdoc	2 to 3 years

53	Non-LAMA/MDP	20	Faculty member	3 years or more
53	Non-LAMA/MDP	20	Faculty member	3 years or more
57	Non-LAMA/MDP	2	Faculty member	1 to 2 years
57	Non-LAMA/MDP	2	Faculty member	3 years or more
58	Non-LAMA/MDP	5	Faculty member	3 years or more
58	Non-LAMA/MDP	5	Faculty member	Less than one year
58	Non-LAMA/MDP	5	Faculty member	3 years or more
58	Non-LAMA/MDP	5	Faculty member	2 to 3 years

Was this mentor assigned to you or someone you found on your own?	What role does this mentor provide for you? (select all that apply)-Research/Scholarly Mentor Provides overall research and/or scholarly guidance.
I found myself	Yes
I found myself	Yes
I found myself	Yes
I found myself	Yes
Other (please explain):	Yes
I found myself	Yes
I found myself	Yes
I found myself	Yes
I found myself	.
I found myself	Yes
I found myself	Yes
Assigned	Yes
I found myself	Yes
Other (please explain):	Yes
Assigned	Yes
I found myself	Yes
Assigned	Yes
Other (please explain):	Yes
I found myself	.
I found myself	Yes
I found myself	Yes
I found myself	Yes
I found myself	Yes
I found myself	Yes
I found myself	.
I found myself	Yes
Other (please explain):	Yes
I found myself	Yes
I found myself	Yes
Other (please explain):	Yes
I found myself	Yes
I found myself	Yes

Assigned	Yes
Other (please explain):	.
Other (please explain):	.
Assigned	Yes
I found myself	.
Other (please explain):	Yes
I found myself	Yes
I found myself	Yes
I found myself	Yes
I found myself	Yes
I found myself	Yes
Other (please explain):	Yes
Assigned	Yes
Assigned	.
Other (please explain):	Yes
Other (please explain):	Yes
I found myself	Yes
I found myself	Yes
I found myself	Yes
I found myself	Yes
I found myself	Yes
Assigned	.
Assigned	.
Assigned	.
I found myself	Yes
Assigned	Yes
I found myself	Yes
Other (please explain):	.
I found myself	Yes
Assigned	.
Other (please explain):	Yes
I found myself	Yes
I found myself	Yes
I found myself	Yes
Other (please explain):	Yes
I found myself	Yes
I found myself	.

Other (please explain):	Yes
I found myself	Yes
Other (please explain):	Yes
I found myself	Yes
I found myself	.
Assigned	.
I found myself	Yes
Assigned	Yes
Assigned	.
Assigned	.
I found myself	Yes
Assigned	.
I found myself	Yes
I found myself	Yes
I found myself	Yes
I found myself	Yes
Other (please explain):	Yes
I found myself	Yes
I found myself	Yes
I found myself	.
I found myself	Yes
Assigned	Yes
Other (please explain):	Yes
Assigned	Yes
I found myself	Yes
I found myself	Yes
I found myself	.
I found myself	Yes
Assigned	Yes
I found myself	Yes
I found myself	Yes

I found myself	Yes
I found myself	Yes
Assigned	.
Assigned	.
I found myself	Yes
I found myself	Yes
I found myself	Yes
I found myself	.
I found myself	Yes
I found myself	.
I found myself	.
I found myself	Yes
I found myself	.
I found myself	Yes
Assigned	Yes
I found myself	.
I found myself	Yes
Other (please explain):	Yes
I found myself	Yes
Assigned	Yes
I found myself	.
Assigned	Yes
I found myself	.
I found myself	Yes
I found myself	Yes
I found myself	Yes
Other (please explain):	Yes
Other (please explain):	Yes
Other (please explain):	Yes
Other (please explain):	.
I found myself	.
Assigned	Yes
Other (please explain):	.
Assigned	Yes
Assigned	Yes
I found myself	Yes
I found myself	.
Assigned	Yes
Assigned	.
I found myself	Yes
I found myself	Yes
I found myself	.
Other (please explain):	.

I found myself	Yes
I found myself	Yes
I found myself	Yes
I found myself	Yes
I found myself	.
I found myself	Yes
I found myself	.
I found myself	Yes

What role does this mentor provide for you? (select all that apply)-Project Mentor Supervises a defined, time-limited project, e.g. data collection, data analysis, manuscript preparation, grant preparation, curriculum or course development.	What role does this mentor provide for you? (select all that apply)-Career Mentor Provides overall career guidance and support mentoring.
.	Yes
.	Yes
Yes	Yes
Yes	Yes
Yes	Yes
.	Yes
Yes	Yes
.	Yes
.	Yes
.	Yes
Yes	Yes
Yes	Yes
Yes	Yes
Yes	Yes
.	Yes
.	Yes
Yes	Yes
.	Yes
.	Yes
Yes	Yes
Yes	Yes
.	Yes
.	Yes
.	Yes
Yes	Yes
.	Yes
.	Yes
Yes	Yes
Yes	Yes
.	Yes
.	Yes
Yes	Yes

Yes	Yes
.	Yes
Yes	Yes
Yes	Yes
.	Yes
Yes	Yes
.	Yes
.	Yes
.	.
.	Yes
.	Yes
.	.
.	Yes
Yes	.
.	Yes
Yes	Yes
Yes	Yes
Yes	Yes
Yes	Yes
.	Yes
.	.
.	Yes
.	Yes
.	Yes
.	Yes
Yes	Yes
Yes	Yes
Yes	Yes
Yes	Yes
.	.
Yes	Yes
Yes	.
.	Yes
Yes	Yes
.	Yes
.	Yes
Yes	Yes

.	Yes
Yes	.
Yes	.
Yes	Yes
Yes	.
Yes	.
.	Yes
Yes	Yes
.	Yes
.	Yes
.	Yes
.	Yes
.	Yes
Yes	Yes
Yes	Yes
.	Yes
.	Yes
.	Yes
.	Yes
Yes	Yes
Yes	Yes
Yes	Yes
Yes	Yes
Yes	Yes
.	.
Yes	Yes
Yes	.
Yes	.
.	Yes
Yes	Yes
.	Yes

Yes	Yes
.	Yes
.	Yes
.	Yes
Yes	Yes
Yes	Yes
.	Yes
Yes	Yes
.	Yes
.	Yes
Yes	Yes
.	Yes
.	Yes
.	Yes
.	Yes
.	Yes
Yes	Yes
Yes	Yes
Yes	Yes
.	Yes
Yes	.
.	.
.	Yes
.	Yes
.	Yes
Yes	.
Yes	Yes
Yes	Yes
Yes	Yes
.	Yes
.	Yes
Yes	Yes
.	Yes
.	Yes
Yes	Yes
Yes	Yes
.	Yes
.	Yes
.	Yes
Yes	Yes
Yes	Yes
Yes	.
Yes	.

.	Yes
Yes	Yes
Yes	.
Yes	Yes
Yes	Yes
.	Yes
.	Yes
Yes	Yes

What role does this mentor provide for you? (select all that apply)-Co-Mentor or Clinical Mentor Provides specialized, content area, methodological or clinical expertise as part of a mentoring research or clinical team.	Please indicate how strongly you agree or disagree with the statements below. - My mentor is accessible.
.	Strongly Agree
Yes	Agree
.	Strongly Agree
.	Strongly Agree
.	Agree
.	Strongly Agree
Yes	Strongly Agree
Yes	Strongly Agree
.	Strongly Agree
.	Strongly Agree
.	Strongly Agree
Yes	Strongly Agree
Yes	Agree
Yes	Neither Disagree or Agree
.	Strongly Agree
.	Strongly Agree
.	Strongly Agree
.	Agree
.	Strongly Agree
.	Strongly Agree
.	Strongly Agree
.	Strongly Agree
.	Strongly Agree
.	Strongly Agree
Yes	Strongly Agree
.	Strongly Agree
.	Strongly Agree
Yes	Strongly Agree
.	Strongly Agree
.	Agree
.	Strongly Agree
.	Agree

.	Agree
.	Strongly Agree
.	Strongly Agree
.	Strongly Agree
.	Agree
Yes	Strongly Agree
.	Strongly Agree
.	Strongly Agree
.	Strongly Agree
Yes	Strongly Agree
.	Strongly Agree
.	Agree
.	Strongly Agree
.	Strongly Agree
.	Agree
Yes	Strongly Agree
Yes	Strongly Agree
Yes	Strongly Agree
.	Strongly Agree
.	Strongly Agree
.	Slightly Agree
.	Agree
.	Agree
Yes	Strongly Agree
.	Strongly Agree
.	Strongly Agree
.	Neither Disagree or Agree
.	Strongly Agree
.	Strongly Agree
Yes	Strongly Agree
.	Strongly Agree
.	Neither Disagree or Agree
Yes	Agree
.	Strongly Agree
.	Strongly Agree
Yes	Strongly Agree
.	Strongly Agree

.	Strongly Agree
.	Agree
.	Strongly Agree
.	Strongly Agree
Yes	Strongly Agree
.	Agree
Yes	Strongly Agree
.	Strongly Agree
.	Strongly Agree
.	Strongly Agree
.	Strongly Agree
.	Agree
.	Strongly Agree
Yes	Strongly Agree
.	Agree
.	Strongly Agree
.	Strongly Agree
.	Strongly Agree
Yes	Strongly Agree
.	Strongly Agree
Yes	Strongly Agree
.	Strongly Agree
.	Strongly Agree
Yes	Disagree
.	Strongly Agree
.	Strongly Agree
.	Agree
.	Strongly Agree
.	Strongly Agree
.	Strongly Agree
Yes	Agree

Yes	Strongly Agree
.	Agree
.	Strongly Agree
.	Agree
.	Strongly Agree
.	Strongly Agree
.	Strongly Agree
.	Slightly Agree
.	Slightly Agree
.	Strongly Agree
Yes	Strongly Agree
Yes	Strongly Agree
.	Strongly Agree
Yes	Strongly Agree
Yes	Strongly Agree
Yes	Strongly Agree
.	Strongly Agree
Yes	Strongly Agree
.	Strongly Agree
.	Strongly Agree
.	Strongly Agree
.	Agree
.	Strongly Agree
.	Strongly Agree
.	Strongly Agree
Yes	Strongly Agree
.	Strongly Agree
.	Strongly Agree
Yes	Strongly Agree
.	Agree
.	Strongly Agree
Yes	Strongly Agree
.	Strongly Agree
.	Strongly Agree
.	Strongly Agree
Yes	Agree
.	Agree
.	Strongly Agree
.	Strongly Agree
Yes	Agree
Yes	Strongly Agree
.	Strongly Agree
Yes	Agree

Yes	Strongly Agree
Yes	Strongly Agree
.	Strongly Disagree
Yes	Strongly Agree
.	Strongly Agree
.	Strongly Agree
.	Strongly Agree
Yes	Strongly Agree

Please indicate how strongly you agree or disagree with the statements below. - My mentor is an active listener.	Please indicate how strongly you agree or disagree with the statements below. - My mentor demonstrates professional expertise.
Strongly Agree	Strongly Agree
Agree	Strongly Agree
Strongly Agree	Strongly Agree
Strongly Agree	Strongly Agree
Strongly Agree	Strongly Agree
Strongly Agree	Strongly Agree
Strongly Agree	Strongly Agree
Strongly Agree	Strongly Agree
Strongly Agree	Strongly Agree
Strongly Agree	Strongly Agree
Strongly Agree	Strongly Agree
Strongly Agree	Strongly Agree
Strongly Agree	Strongly Agree
Agree	Strongly Agree
Strongly Agree	Strongly Agree
Strongly Agree	Strongly Agree
Strongly Agree	Strongly Agree
Strongly Agree	Strongly Agree
Strongly Agree	Strongly Agree
Strongly Agree	Strongly Agree
Strongly Agree	Strongly Agree
Strongly Agree	Strongly Agree
Strongly Agree	Strongly Agree
Strongly Agree	Strongly Agree
Strongly Agree	Strongly Agree
Agree	Strongly Agree
Strongly Agree	Strongly Agree
Strongly Agree	Strongly Agree
Strongly Agree	Strongly Agree
Agree	Agree
Agree	Strongly Agree
Strongly Agree	Strongly Agree

Neither Disagree or Agree	Strongly Agree
Agree	Strongly Agree
Strongly Agree	Strongly Agree
Strongly Agree	Strongly Agree
Agree	Strongly Agree
Strongly Agree	Strongly Agree
Strongly Agree	Strongly Agree
Strongly Agree	Strongly Agree
Strongly Agree	Strongly Agree
Strongly Agree	Strongly Agree
Strongly Agree	Strongly Agree
Agree	Agree
Strongly Agree	Strongly Agree
Strongly Agree	Strongly Agree
Agree	Strongly Agree
Strongly Agree	Strongly Agree
Strongly Agree	Strongly Agree
Strongly Agree	Strongly Agree
Strongly Agree	Strongly Agree
Strongly Agree	Strongly Agree
Strongly Agree	Strongly Agree
Strongly Agree	Strongly Agree
Strongly Agree	Agree
Strongly Agree	Strongly Agree
Strongly Agree	Strongly Agree
Strongly Agree	Strongly Agree
Slightly Agree	Agree
Agree	Strongly Agree
Strongly Agree	Strongly Agree
Strongly Agree	Strongly Agree
Strongly Agree	Strongly Agree
Agree	Strongly Agree
Agree	Strongly Agree
Strongly Agree	Strongly Agree
Strongly Agree	Strongly Agree
Strongly Agree	Strongly Agree
Strongly Agree	Strongly Agree

Agree	Strongly Agree
Agree	Strongly Agree
Agree	Strongly Agree
Strongly Agree	Strongly Agree
Strongly Agree	Strongly Agree
Agree	Agree
Strongly Agree	Strongly Agree
Strongly Agree	Strongly Agree
Strongly Agree	Strongly Agree
Strongly Agree	Strongly Agree
Strongly Agree	Strongly Agree
Agree	Strongly Agree
Strongly Agree	Strongly Agree
Strongly Agree	Strongly Agree
Agree	Strongly Agree
Strongly Agree	Strongly Agree
Strongly Agree	Strongly Agree
Agree	Agree
Strongly Agree	Strongly Agree
Strongly Agree	Strongly Agree
Agree	Strongly Agree
Strongly Agree	Strongly Agree
Strongly Agree	Strongly Agree
Agree	Agree
Strongly Agree	Strongly Agree
Agree	Strongly Agree
Strongly Agree	Strongly Agree
Agree	Strongly Agree
Agree	Strongly Agree
Strongly Agree	Strongly Agree
Agree	Agree

Strongly Agree	Strongly Agree
Agree	Strongly Agree
Strongly Agree	Strongly Agree
Agree	Agree
Strongly Agree	Strongly Agree
Strongly Agree	Strongly Agree
Strongly Agree	Strongly Agree
Neither Disagree or Agree	Slightly Agree
Agree	Strongly Agree
Strongly Agree	Strongly Agree
Strongly Agree	Strongly Agree
Strongly Agree	Strongly Agree
Strongly Agree	Strongly Agree
Strongly Agree	Strongly Agree
Strongly Agree	Strongly Agree
Strongly Agree	Strongly Agree
Strongly Agree	Strongly Agree
Strongly Agree	Strongly Agree
Strongly Agree	Strongly Agree
Strongly Agree	Strongly Agree
Strongly Agree	Strongly Agree
Agree	Agree
Strongly Agree	Strongly Agree
Strongly Agree	Strongly Agree
Strongly Agree	Strongly Agree
Agree	Strongly Agree
Strongly Agree	Strongly Agree
Strongly Agree	Strongly Agree
Strongly Agree	Strongly Agree
Strongly Agree	Strongly Agree
Agree	Strongly Agree
Strongly Agree	Strongly Agree
Strongly Agree	Strongly Agree
Strongly Agree	Strongly Agree
Strongly Agree	Strongly Agree
Strongly Agree	Strongly Agree
Strongly Agree	Strongly Agree
Strongly Agree	Strongly Agree
Strongly Agree	Strongly Agree
Agree	Strongly Agree
Strongly Agree	Strongly Agree
Strongly Agree	Strongly Agree
Strongly Agree	Strongly Agree

Agree	Strongly Agree
Strongly Agree	Strongly Agree
Strongly Disagree	Strongly Disagree
Strongly Agree	Strongly Agree
Strongly Agree	Strongly Agree
Strongly Agree	Strongly Agree
Strongly Agree	Strongly Agree
Agree	Strongly Agree

Please indicate how strongly you agree or disagree with the statements below. - My mentor encourages me to establish an independent career.	Please indicate how strongly you agree or disagree with the statements below. -My mentor provides useful critiques of my work.
Strongly Agree	Agree
Strongly Agree	Agree
Strongly Agree	Agree
Strongly Agree	Strongly Agree
Strongly Agree	Strongly Agree
Strongly Agree	Strongly Agree
Strongly Agree	Strongly Agree
Strongly Agree	Strongly Agree
Strongly Agree	.
Strongly Agree	Strongly Agree
Strongly Agree	Strongly Agree
Strongly Agree	Strongly Agree
Strongly Agree	Strongly Agree
Strongly Agree	Agree
Strongly Agree	Strongly Agree
Strongly Agree	Strongly Agree
Strongly Agree	Strongly Agree
Strongly Agree	Strongly Agree
Strongly Agree	Strongly Agree
Strongly Agree	Agree
Strongly Agree	Strongly Agree
Strongly Agree	Strongly Agree
Agree	Agree
Strongly Agree	Strongly Agree
Strongly Agree	Strongly Agree
Strongly Agree	Strongly Agree
Strongly Agree	Strongly Agree
Agree	Agree
Strongly Agree	Strongly Agree
Agree	Agree
Agree	Neither Disagree or Agree
Strongly Agree	Slightly Disagree

Agree	Agree
Strongly Agree	Strongly Agree
Strongly Agree	Strongly Agree
Strongly Agree	Strongly Agree
Strongly Agree	Slightly Agree
Strongly Agree	Strongly Agree
Strongly Agree	Strongly Agree
Strongly Agree	Strongly Agree
Strongly Agree	Strongly Agree
Strongly Agree	Strongly Agree
Strongly Agree	Strongly Agree
Agree	Agree
Strongly Agree	Strongly Agree
Strongly Agree	Strongly Agree
Strongly Agree	Strongly Agree
Strongly Agree	Strongly Agree
Strongly Agree	Strongly Agree
Strongly Agree	Strongly Agree
Strongly Agree	Strongly Agree
Strongly Agree	Strongly Agree
Strongly Agree	Strongly Agree
Strongly Agree	Strongly Agree
Agree	Agree
Strongly Agree	Strongly Agree
Strongly Agree	Strongly Agree
Strongly Agree	Strongly Agree
Slightly Agree	Neither Disagree or Agree
Strongly Agree	Strongly Agree
Strongly Agree	Strongly Agree
Strongly Agree	Slightly Agree
Strongly Agree	Strongly Agree
Agree	Strongly Agree
Agree	Agree
Strongly Agree	Strongly Agree
Strongly Agree	Strongly Agree
Strongly Agree	Strongly Agree
Agree	Agree

Agree	Strongly Agree
Agree	Strongly Agree
Agree	Strongly Agree
Strongly Agree	Strongly Agree
.	Agree
.	Agree
Strongly Agree	Strongly Agree
Strongly Agree	Strongly Agree
Strongly Agree	.
Strongly Agree	.
Strongly Agree	Strongly Agree
Agree	.
Strongly Agree	Strongly Agree
Strongly Agree	Agree
Strongly Agree	Strongly Agree
Strongly Agree	Strongly Agree
Strongly Agree	Strongly Agree
Strongly Agree	Agree
Strongly Agree	Strongly Agree
Strongly Agree	Agree
Strongly Agree	Agree
Strongly Agree	Strongly Agree
Strongly Agree	Strongly Agree
Neither Disagree or Agree	Strongly Disagree
Strongly Agree	Strongly Agree
Agree	Strongly Agree
Strongly Agree	Agree
Agree	Agree
Strongly Agree	Strongly Agree
Strongly Agree	Strongly Agree
Strongly Agree	Agree

Agree	Agree
Strongly Agree	Agree
Agree	Slightly Agree
Agree	Slightly Agree
Strongly Agree	Strongly Agree
Strongly Agree	Strongly Agree
Strongly Agree	Strongly Agree
Strongly Agree	Neither Disagree or Agree
Strongly Agree	Slightly Agree
Strongly Agree	Slightly Agree
Strongly Agree	Strongly Agree
Strongly Agree	Strongly Agree
Strongly Agree	Strongly Agree
Agree	Agree
Strongly Agree	Agree
Strongly Agree	.
Strongly Agree	Strongly Agree
.	Strongly Agree
Strongly Agree	Strongly Agree
Strongly Agree	Strongly Agree
Strongly Agree	Strongly Agree
Agree	Agree
Strongly Agree	Agree
Strongly Agree	.
Strongly Agree	Strongly Agree
Strongly Agree	Strongly Agree
Strongly Agree	Strongly Agree
Strongly Agree	Strongly Agree
Strongly Agree	Strongly Agree
Strongly Agree	Strongly Agree
Strongly Agree	Strongly Agree
Strongly Agree	Agree
Strongly Agree	Strongly Agree
Strongly Agree	Agree
Strongly Agree	Agree
Strongly Agree	Agree
Strongly Agree	Strongly Agree
Strongly Agree	Strongly Agree
Strongly Agree	Strongly Agree
Strongly Agree	Agree
Strongly Agree	Strongly Agree
Strongly Agree	Strongly Agree
Strongly Agree	Strongly Agree

Agree	Agree
Strongly Agree	Strongly Agree
Strongly Disagree	Strongly Disagree
Strongly Agree	Strongly Agree
Strongly Agree	Agree
Strongly Agree	Strongly Agree
Strongly Agree	Strongly Agree
Agree	Agree

Please indicate how strongly you agree or disagree with the statements below. -My mentor motivates me to improve my work.	Please indicate how strongly you agree or disagree with the statements below. -My mentor is helpful in providing direction and guidance on professional issues.
Strongly Agree	Strongly Agree
Agree	Agree
Agree	Strongly Agree
Strongly Agree	Strongly Agree
Strongly Agree	Strongly Agree
Strongly Agree	Strongly Agree
Strongly Agree	Strongly Agree
Strongly Agree	Strongly Agree
Strongly Agree	Strongly Agree
Strongly Agree	Strongly Agree
Strongly Agree	Strongly Agree
Strongly Agree	Strongly Agree
Agree	Strongly Agree
Strongly Agree	Strongly Agree
.	Strongly Agree
Strongly Agree	Strongly Agree
Strongly Agree	Strongly Agree
Strongly Agree	Agree
Agree	Strongly Agree
Agree	Strongly Agree
Strongly Agree	Strongly Agree
Strongly Agree	Strongly Agree
Agree	Strongly Agree
Strongly Agree	Strongly Agree
Strongly Agree	Strongly Agree
Strongly Agree	Strongly Agree
Strongly Agree	Strongly Agree
Strongly Agree	Agree
Strongly Agree	Strongly Agree
Agree	Agree
Slightly Agree	Agree
Slightly Agree	Agree

Agree	Agree
Agree	Strongly Agree
Strongly Agree	Strongly Agree
Strongly Agree	Strongly Agree
Slightly Agree	Strongly Agree
Strongly Agree	Strongly Agree
Strongly Agree	Strongly Agree
Strongly Agree	Strongly Agree
Strongly Agree	Strongly Agree
Strongly Agree	Strongly Agree
Strongly Agree	Strongly Agree
Agree	Agree
Strongly Agree	Strongly Agree
Strongly Agree	Strongly Agree
Strongly Agree	Agree
Strongly Agree	Agree
Strongly Agree	Strongly Agree
Strongly Agree	Strongly Agree
Strongly Agree	Strongly Agree
Strongly Agree	Strongly Agree
Strongly Agree	Agree
Agree	Strongly Agree
Agree	Agree
Strongly Agree	Strongly Agree
Strongly Agree	Strongly Agree
Strongly Agree	Strongly Agree
Slightly Agree	Neither Disagree or Agree
Agree	Agree
Strongly Agree	Strongly Agree
Agree	Strongly Agree
Strongly Agree	Strongly Agree
Agree	Slightly Agree
Strongly Agree	Strongly Agree
Strongly Agree	Strongly Agree
Strongly Agree	Strongly Agree
Strongly Agree	Strongly Agree
Agree	Agree

Strongly Agree	Strongly Agree
Strongly Agree	Agree
Strongly Agree	Agree
Strongly Agree	Strongly Agree
Agree	Slightly Agree
Agree	Agree
Strongly Agree	Strongly Agree
Strongly Agree	Strongly Agree
.	Agree
Strongly Agree	Strongly Agree
Strongly Agree	Strongly Agree
.	Agree
Strongly Agree	Strongly Agree
Strongly Agree	Strongly Agree
Slightly Agree	Strongly Agree
Strongly Agree	Agree
Strongly Agree	Strongly Agree
Slightly Agree	Agree
Strongly Agree	Strongly Agree
Agree	Strongly Agree
Agree	Slightly Agree
Strongly Agree	Strongly Agree
Strongly Agree	Strongly Agree
Strongly Disagree	Neither Disagree or Agree
Strongly Agree	Strongly Agree
Strongly Agree	Strongly Agree
Agree	Agree
Agree	Agree
Strongly Agree	Agree
Strongly Agree	Strongly Agree
Agree	Agree

Agree	Strongly Agree
Strongly Agree	Strongly Agree
Agree	Agree
Slightly Agree	Agree
Strongly Agree	Strongly Agree
Strongly Agree	Strongly Agree
Strongly Agree	Strongly Agree
Neither Disagree or Agree	Neither Disagree or Agree
Agree	Slightly Agree
Strongly Agree	Strongly Agree
Strongly Agree	Strongly Agree
Strongly Agree	Strongly Agree
Strongly Agree	Strongly Agree
Agree	Strongly Agree
Agree	Strongly Agree
Agree	Strongly Agree
Strongly Agree	Strongly Agree
Agree	Strongly Agree
Strongly Agree	Strongly Agree
Strongly Agree	Strongly Agree
Agree	Strongly Agree
Agree	Agree
Agree	Strongly Agree
Strongly Agree	Strongly Agree
Strongly Agree	Strongly Agree
Strongly Agree	Strongly Agree
Strongly Agree	Strongly Agree
Strongly Agree	Strongly Agree
Strongly Agree	Strongly Agree
Strongly Agree	Slightly Agree
Agree	Agree
Agree	Strongly Agree
Strongly Agree	Strongly Agree
Strongly Agree	Agree
Agree	Strongly Agree
Slightly Agree	Agree
Strongly Agree	Strongly Agree
Strongly Agree	Strongly Agree
Strongly Agree	Strongly Agree
Strongly Agree	Strongly Agree
Strongly Agree	Strongly Agree
Strongly Agree	Strongly Agree
Strongly Agree	.

Strongly Agree	Strongly Agree
Strongly Agree	Strongly Agree
Strongly Disagree	Strongly Disagree
Strongly Agree	Strongly Agree
Agree	Strongly Agree
Strongly Agree	Strongly Agree
Agree	Agree
Strongly Agree	Agree

Please indicate how strongly you agree or disagree with the statements below. -My mentor acknowledges my contributions appropriately.	Please indicate how strongly you agree or disagree with the statements below. -My mentor takes a sincere interest in my career.
Strongly Agree	Strongly Agree
Strongly Agree	Strongly Agree
Strongly Agree	Strongly Agree
Strongly Agree	Strongly Agree
Strongly Agree	Strongly Agree
Strongly Agree	Strongly Agree
Strongly Agree	Strongly Agree
Strongly Agree	Strongly Agree
.	Agree
Strongly Agree	Strongly Agree
Strongly Agree	Strongly Agree
Strongly Agree	Strongly Agree
Strongly Agree	Strongly Agree
Strongly Agree	Strongly Agree
.	Strongly Agree
Strongly Agree	Strongly Agree
Strongly Agree	Strongly Agree
Strongly Agree	Strongly Agree
Strongly Agree	Strongly Agree
Agree	Agree
Strongly Agree	Strongly Agree
Strongly Agree	Strongly Agree
Agree	Strongly Agree
Strongly Agree	Strongly Agree
Strongly Agree	Strongly Agree
Strongly Agree	Strongly Agree
Strongly Agree	Strongly Agree
Agree	Strongly Agree
Strongly Agree	Strongly Agree
Agree	Agree
Strongly Agree	Strongly Agree
Strongly Agree	Strongly Agree

Agree	Strongly Agree
Strongly Agree	Strongly Agree
Strongly Agree	Strongly Agree
Strongly Agree	Strongly Agree
Agree	Strongly Agree
Strongly Agree	Strongly Agree
Strongly Agree	Strongly Agree
Strongly Agree	Strongly Agree
Strongly Agree	Strongly Agree
Strongly Agree	Strongly Agree
Strongly Agree	Strongly Agree
Agree	Agree
Strongly Agree	Strongly Agree
Strongly Agree	Strongly Agree
Strongly Agree	Strongly Agree
Strongly Agree	Strongly Agree
Strongly Agree	Strongly Agree
Strongly Agree	Strongly Agree
Strongly Agree	Strongly Agree
Strongly Agree	Strongly Agree
Agree	Agree
Strongly Agree	Strongly Agree
Agree	Strongly Agree
Strongly Agree	Strongly Agree
Strongly Agree	Strongly Agree
Strongly Agree	Strongly Agree
Slightly Agree	Neither Disagree or Agree
Strongly Agree	Strongly Agree
Strongly Agree	Strongly Agree
Agree	Strongly Agree
Strongly Agree	Strongly Agree
Strongly Agree	Strongly Agree
Strongly Agree	Agree
Strongly Agree	Strongly Agree
Strongly Agree	Strongly Agree
Strongly Agree	Strongly Agree
Strongly Agree	Slightly Agree

Agree	Agree
Strongly Agree	Agree
Agree	Agree
Strongly Agree	Strongly Agree
Agree	Slightly Agree
Agree	Agree
Strongly Agree	Strongly Agree
Strongly Agree	Strongly Agree
.	Agree
Strongly Agree	Strongly Agree
Strongly Agree	Strongly Agree
.	Agree
Strongly Agree	Strongly Agree
Strongly Agree	Strongly Agree
Strongly Agree	Strongly Agree
Strongly Agree	Strongly Agree
Strongly Agree	Strongly Agree
Strongly Agree	Strongly Agree
Agree	Strongly Agree
Strongly Agree	Strongly Agree
Agree	Strongly Agree
Strongly Agree	Strongly Agree
Strongly Agree	Strongly Agree
Agree	Disagree
Strongly Agree	Strongly Agree
Strongly Agree	Strongly Agree
Strongly Agree	Strongly Agree
Agree	Strongly Agree
Strongly Agree	Strongly Agree
Strongly Agree	Strongly Agree
Agree	Strongly Agree

Agree	Agree
Strongly Agree	Strongly Agree
Strongly Agree	Strongly Agree
Agree	Agree
Agree	Strongly Agree
Strongly Agree	Strongly Agree
Strongly Agree	Strongly Agree
Neither Disagree or Agree	Slightly Agree
Strongly Agree	Strongly Agree
Agree	Strongly Agree
Strongly Agree	Strongly Agree
Strongly Agree	Strongly Agree
Strongly Agree	Strongly Agree
Strongly Agree	Strongly Agree
Strongly Agree	Strongly Agree
Strongly Agree	Strongly Agree
Strongly Agree	Strongly Agree
Strongly Agree	Strongly Agree
Strongly Agree	Strongly Agree
Strongly Agree	Strongly Agree
Strongly Agree	Strongly Agree
Agree	Agree
Strongly Agree	Strongly Agree
.	Strongly Agree
Strongly Agree	Strongly Agree
Strongly Agree	Strongly Agree
Strongly Agree	Strongly Agree
Strongly Agree	Strongly Agree
Strongly Agree	Strongly Agree
Agree	Strongly Agree
Strongly Agree	Strongly Agree
Strongly Agree	Strongly Agree
Strongly Agree	Strongly Agree
Strongly Agree	Strongly Agree
Strongly Agree	Strongly Agree
Strongly Agree	Strongly Agree
Strongly Agree	Strongly Agree
Strongly Agree	Strongly Agree
Strongly Agree	Strongly Agree
Strongly Agree	Strongly Agree
Strongly Agree	Strongly Agree
Strongly Agree	Strongly Agree
Strongly Agree	Strongly Agree

Strongly Agree	Strongly Agree
Strongly Agree	Strongly Agree
Strongly Disagree	Strongly Disagree
Strongly Agree	Strongly Agree
Strongly Agree	Strongly Agree
Strongly Agree	Strongly Agree
Strongly Agree	Strongly Agree
Strongly Agree	Strongly Agree

Please indicate how strongly you agree or disagree with the statements below. -My mentor helps me to formulate clear goals.	Please indicate how strongly you agree or disagree with the statements below. -My mentor facilitates building my professional network.
Agree	Strongly Agree
Agree	Strongly Agree
Agree	Strongly Agree
Strongly Agree	Strongly Agree
Agree	Strongly Agree
Agree	Strongly Agree
Strongly Agree	Strongly Agree
Strongly Agree	Strongly Agree
Agree	Agree
Strongly Agree	Strongly Agree
Strongly Agree	Agree
Strongly Agree	Strongly Agree
Strongly Agree	Strongly Agree
Agree	Agree
Strongly Agree	.
Strongly Agree	Strongly Agree
Strongly Agree	Strongly Agree
Agree	Agree
Agree	Agree
Agree	Slightly Agree
Strongly Agree	Strongly Agree
Strongly Agree	Strongly Agree
Agree	Agree
Strongly Agree	Strongly Agree
Agree	Strongly Agree
Strongly Agree	Strongly Agree
Strongly Agree	Strongly Agree
Agree	Agree
Strongly Agree	Strongly Agree
Agree	Agree
Agree	Agree
Neither Disagree or Agree	Agree

Agree	Agree
Agree	Strongly Agree
Strongly Agree	Strongly Agree
Strongly Agree	Strongly Agree
Strongly Agree	Strongly Agree
Strongly Agree	Strongly Agree
Strongly Agree	Strongly Agree
Strongly Agree	Strongly Agree
Agree	Agree
Strongly Agree	Slightly Agree
Strongly Agree	Strongly Agree
Agree	Agree
Agree	Agree
Strongly Agree	Agree
Strongly Agree	Strongly Agree
Strongly Agree	Strongly Agree
Strongly Agree	Strongly Agree
Strongly Agree	Strongly Agree
Strongly Agree	Strongly Agree
Strongly Agree	Strongly Agree
Strongly Agree	Agree
Strongly Agree	Agree
Agree	Agree
Strongly Agree	Strongly Agree
Slightly Agree	Slightly Agree
Strongly Agree	Strongly Agree
Agree	Slightly Disagree
Agree	Strongly Agree
Strongly Agree	Strongly Agree
Strongly Agree	Strongly Agree
Strongly Agree	Strongly Agree
Agree	Agree
Agree	Agree
Strongly Agree	Strongly Agree
Strongly Agree	Strongly Agree
Strongly Agree	Strongly Agree
Agree	Neither Disagree or Agree

Agree	Agree
Agree	Agree
Strongly Agree	.
Strongly Agree	Strongly Agree
Agree	Agree
.	.
Strongly Agree	Strongly Agree
Strongly Agree	.
Agree	Agree
Strongly Agree	.
Strongly Agree	Strongly Agree
Agree	Agree
Agree	Slightly Agree
Strongly Agree	Agree
Slightly Agree	Strongly Agree
Strongly Agree	Slightly Agree
Strongly Agree	Strongly Agree
Strongly Agree	Strongly Agree
Strongly Agree	Agree
Strongly Agree	Strongly Agree
Slightly Agree	Neither Disagree or Agree
Strongly Agree	Strongly Agree
Strongly Agree	Strongly Agree
Neither Disagree or Agree	Disagree
Strongly Agree	Strongly Agree
Agree	Strongly Agree
Strongly Agree	Strongly Agree
Strongly Agree	Strongly Agree
Slightly Agree	Agree
Strongly Agree	Strongly Agree
Agree	Agree

Agree	Strongly Agree
Strongly Agree	Strongly Agree
Agree	Agree
Slightly Agree	Agree
Strongly Agree	Strongly Agree
Strongly Agree	Strongly Agree
Strongly Agree	Agree
Neither Disagree or Agree	Neither Disagree or Agree
Agree	Strongly Agree
Strongly Agree	Slightly Agree
Strongly Agree	Strongly Agree
Strongly Agree	Strongly Agree
Strongly Agree	Strongly Agree
Strongly Agree	Agree
Strongly Agree	Strongly Agree
Strongly Agree	Strongly Agree
Strongly Agree	Strongly Agree
Agree	Agree
Strongly Agree	Strongly Agree
Strongly Agree	Strongly Agree
Strongly Agree	Strongly Agree
Agree	Agree
Strongly Agree	Strongly Agree
Strongly Agree	Strongly Agree
Strongly Agree	Strongly Agree
Agree	Agree
Strongly Agree	Strongly Agree
Strongly Agree	Strongly Agree
Strongly Agree	Strongly Agree
Agree	Slightly Agree
Strongly Agree	Slightly Agree
Agree	Agree
Strongly Agree	Strongly Agree
Strongly Agree	Agree
Strongly Agree	Strongly Agree
Agree	Agree
Agree	Slightly Agree
Strongly Agree	Strongly Agree
Agree	Agree
Agree	Strongly Agree
Strongly Agree	Strongly Agree
Strongly Agree	Strongly Agree
Strongly Agree	Agree

Agree	Strongly Agree
Strongly Agree	Strongly Agree
Strongly Disagree	Strongly Disagree
Strongly Agree	Strongly Agree
Strongly Agree	Agree
Strongly Agree	Strongly Agree
Slightly Agree	Strongly Agree
Agree	Agree

Please indicate how strongly you agree or disagree with the statements below. -My mentor provides thoughtful advice on my scholarly work.	Please indicate how strongly you agree or disagree with the statements below. -My mentor is supportive of work-life balance.
Strongly Agree	Agree
Agree	Strongly Agree
Agree	Agree
Strongly Agree	Strongly Agree
Strongly Agree	Strongly Agree
Strongly Agree	Strongly Agree
Strongly Agree	Strongly Agree
Strongly Agree	Strongly Agree
.	Strongly Agree
Strongly Agree	.
Strongly Agree	Agree
Strongly Agree	Strongly Agree
Strongly Agree	Strongly Agree
Strongly Agree	Strongly Agree
Strongly Agree	.
Strongly Agree	Strongly Agree
Strongly Agree	Strongly Agree
Strongly Agree	Strongly Agree
Agree	Strongly Agree
Agree	Agree
Strongly Agree	Strongly Agree
Strongly Agree	Strongly Agree
Strongly Agree	Strongly Agree
Strongly Agree	Strongly Agree
Strongly Agree	Agree
Strongly Agree	Slightly Agree
Strongly Agree	Slightly Agree
Strongly Agree	Agree
Strongly Agree	Strongly Agree
Slightly Agree	Strongly Agree
Neither Disagree or Agree	Strongly Agree
Slightly Disagree	Agree

Agree	Neither Disagree or Agree
Strongly Agree	Strongly Agree
.	.
Strongly Agree	Strongly Agree
Agree	Strongly Agree
Strongly Agree	Strongly Agree
Strongly Agree	Strongly Agree
Strongly Agree	Strongly Agree
Strongly Agree	Strongly Agree
Strongly Agree	Strongly Agree
Strongly Agree	Strongly Agree
Agree	Agree
Agree	Strongly Agree
Strongly Agree	Agree
Strongly Agree	Strongly Agree
Strongly Agree	Agree
Strongly Agree	Strongly Agree
Strongly Agree	Strongly Agree
Strongly Agree	Strongly Agree
Strongly Agree	Strongly Agree
Strongly Agree	Strongly Agree
.	Agree
Agree	Agree
Strongly Agree	Strongly Agree
Strongly Agree	Strongly Agree
Strongly Agree	Strongly Agree
Neither Disagree or Agree	Neither Disagree or Agree
Strongly Agree	Strongly Agree
Strongly Agree	Strongly Agree
Strongly Agree	Agree
Strongly Agree	Strongly Agree
Agree	Strongly Agree
Agree	Agree
Strongly Agree	Strongly Agree
Strongly Agree	Strongly Agree
Strongly Agree	Strongly Agree
Strongly Agree	Neither Disagree or Agree

Agree	Agree
Strongly Agree	Strongly Agree
Strongly Agree	.
Strongly Agree	Strongly Agree
Strongly Agree	.
Agree	Agree
Strongly Agree	Strongly Agree
Strongly Agree	Strongly Agree
Agree	Agree
.	Strongly Agree
Strongly Agree	Strongly Agree
.	.
Agree	Strongly Agree
Strongly Agree	Strongly Agree
Agree	Strongly Agree
Strongly Agree	Agree
Strongly Agree	Strongly Agree
Slightly Agree	Strongly Agree
Agree	Agree
Strongly Agree	Strongly Agree
Agree	Agree
Strongly Agree	Strongly Agree
Strongly Agree	Strongly Agree
Strongly Disagree	.
Strongly Agree	Strongly Agree
Strongly Agree	Agree
Agree	.
Strongly Agree	Strongly Agree
Strongly Agree	Agree
Strongly Agree	Strongly Agree
Agree	Agree

Agree	Strongly Agree
Strongly Agree	Agree
Slightly Agree	Strongly Agree
.	Agree
Strongly Agree	Strongly Agree
Strongly Agree	Strongly Agree
Agree	Strongly Agree
Neither Disagree or Agree	Agree
Agree	Strongly Agree
Strongly Agree	Strongly Agree
Strongly Agree	Strongly Agree
Strongly Agree	Strongly Agree
Strongly Agree	Strongly Agree
Strongly Agree	Strongly Agree
Strongly Agree	Strongly Agree
.	Strongly Agree
Strongly Agree	Strongly Agree
Strongly Agree	Strongly Agree
Strongly Agree	Strongly Agree
Strongly Agree	Strongly Agree
Strongly Agree	Strongly Agree
Agree	Agree
Strongly Agree	Strongly Agree
Strongly Agree	Strongly Agree
Strongly Agree	Strongly Agree
Strongly Agree	Slightly Agree
Strongly Agree	Strongly Agree
Strongly Agree	Agree
Strongly Agree	Strongly Agree
Agree	Strongly Agree
Strongly Agree	Strongly Agree
Strongly Agree	Strongly Agree
.	Strongly Agree
Agree	Strongly Agree
Strongly Agree	Strongly Agree
Agree	Strongly Agree
Neither Disagree or Agree	Strongly Agree
Strongly Agree	Strongly Agree
Strongly Agree	Strongly Agree
Strongly Agree	Strongly Agree
Strongly Agree	Strongly Agree
Strongly Agree	Strongly Agree
Strongly Agree	Strongly Agree

Strongly Agree	Agree
Strongly Agree	Strongly Agree
Strongly Disagree	Strongly Disagree
Strongly Agree	Strongly Agree
Agree	Strongly Agree
Strongly Agree	Strongly Agree
Agree	Strongly Agree
Agree	Agree

Please indicate how strongly you agree or disagree with the statements below. - Overall, I am satisfied with my mentor.	Mean of Q10a-Q10m	Dichotomized-How many years has this person been your mentor?	nmiss
Strongly Agree	6.77	Greater than 3 years	0
Strongly Agree	6.46	Greater than 3 years	0
Strongly Agree	6.62	Greater than 3 years	0
Strongly Agree	7	Greater than 3 years	0
Strongly Agree	6.85	Greater than 3 years	0
Strongly Agree	6.92	Greater than 3 years	0
Strongly Agree	7	Greater than 3 years	0
Strongly Agree	7	Greater than 3 years	0
Strongly Agree	6.7	Greater than 3 years	3
Strongly Agree	7	Greater than 3 years	1
Strongly Agree	6.85	Less than three years	0
Strongly Agree	7	Greater than 3 years	0
Strongly Agree	6.85	Greater than 3 years	0
Strongly Agree	6.46	Greater than 3 years	0
Strongly Agree	7	Greater than 3 years	4
Strongly Agree	7	Greater than 3 years	0
Strongly Agree	7	Greater than 3 years	0
Strongly Agree	6.69	Greater than 3 years	0
Strongly Agree	6.69	Greater than 3 years	0
Agree	6.31	Greater than 3 years	0
Strongly Agree	7	Greater than 3 years	0
Strongly Agree	7	Greater than 3 years	0
Strongly Agree	6.54	Less than three years	0
Strongly Agree	7	Greater than 3 years	0
Strongly Agree	6.85	Greater than 3 years	0
Strongly Agree	6.77	Greater than 3 years	0
Strongly Agree	6.85	Greater than 3 years	0
Strongly Agree	6.46	Greater than 3 years	0
Strongly Agree	7	Greater than 3 years	0
Strongly Agree	6	Greater than 3 years	0
Agree	6	Less than three years	0
Agree	5.69	Less than three years	0

Agree	5.85	Greater than 3 years	0
Strongly Agree	6.77	Greater than 3 years	0
Strongly Agree	7	Greater than 3 years	2
Strongly Agree	7	Less than three years	0
Strongly Agree	6.38	Greater than 3 years	0
Strongly Agree	7	Greater than 3 years	0
Strongly Agree	7	Greater than 3 years	0
Strongly Agree	7	Greater than 3 years	0
Strongly Agree	6.85	Greater than 3 years	0
Strongly Agree	6.85	Greater than 3 years	0
Strongly Agree	7	Greater than 3 years	0
Agree	6	Less than three years	0
Strongly Agree	6.77	Greater than 3 years	0
Strongly Agree	6.85	Greater than 3 years	0
Strongly Agree	6.77	Greater than 3 years	0
Strongly Agree	6.85	Less than three years	0
Strongly Agree	7	Greater than 3 years	0
Strongly Agree	7	Greater than 3 years	0
Strongly Agree	7	Greater than 3 years	0
Strongly Agree	7	Greater than 3 years	0
Strongly Agree	6.54	Less than three years	0
Strongly Agree	6.67	Less than three years	1
Agree	6.15	Less than three years	0
Strongly Agree	7	Less than three years	0
Strongly Agree	6.69	Less than three years	0
Strongly Agree	7	Greater than 3 years	0
Slightly Agree	4.54	Less than three years	0
Strongly Agree	6.69	Greater than 3 years	0
Strongly Agree	7	Greater than 3 years	0
Strongly Agree	6.62	Less than three years	0
Strongly Agree	7	Greater than 3 years	0
Agree	6.15	Greater than 3 years	0
Strongly Agree	6.31	Greater than 3 years	0
Strongly Agree	7	Greater than 3 years	0
Strongly Agree	7	Greater than 3 years	0
Strongly Agree	7	Greater than 3 years	0
Strongly Agree	6	Less than three years	0

Agree	6.38	Less than three years	0
Strongly Agree	6.46	Less than three years	0
Agree	6.55	Greater than 3 years	2
Strongly Agree	7	Less than three years	0
Strongly Agree	6.18	Greater than 3 years	2
Agree	6	Less than three years	3
Strongly Agree	7	Greater than 3 years	0
Strongly Agree	7	Less than three years	1
Agree	6.4	Less than three years	3
Strongly Agree	7	Less than three years	3
Strongly Agree	7	Greater than 3 years	0
Strongly Agree	6.13	Less than three years	5
Strongly Agree	6.69	Greater than 3 years	0
Strongly Agree	6.85	Greater than 3 years	0
Slightly Agree	6.46	Greater than 3 years	0
Strongly Agree	6.69	Greater than 3 years	0
Strongly Agree	7	Greater than 3 years	0
Strongly Agree	6.38	Greater than 3 years	0
Strongly Agree	6.69	Less than three years	0
Strongly Agree	6.85	Less than three years	0
Agree	6	Less than three years	0
Strongly Agree	7	Less than three years	0
Strongly Agree	7	Greater than 3 years	0
Disagree	3.25	Less than three years	1
Strongly Agree	7	Less than three years	0
Strongly Agree	6.69	Less than three years	0
Strongly Agree	6.58	Less than three years	1
Strongly Agree	6.54	Less than three years	0
Strongly Agree	6.54	Greater than 3 years	0
Strongly Agree	7	Less than three years	0
Agree	6.15	Less than three years	0

Strongly Agree	6.46	Greater than 3 years	0
Strongly Agree	6.69	Greater than 3 years	0
Agree	6.31	Less than three years	0
Slightly Agree	5.75	Greater than 3 years	1
Strongly Agree	6.92	Greater than 3 years	0
Strongly Agree	7	Greater than 3 years	0
Strongly Agree	6.85	Greater than 3 years	0
Neither Disagree or Agree	4.62	Greater than 3 years	0
Strongly Agree	6.23	Greater than 3 years	0
Strongly Agree	6.62	Less than three years	0
Strongly Agree	7	Less than three years	0
Strongly Agree	7	Less than three years	0
Strongly Agree	7	Greater than 3 years	0
Strongly Agree	6.69	Greater than 3 years	0
Strongly Agree	6.85	Less than three years	0
Strongly Agree	6.91	Greater than 3 years	2
Strongly Agree	7	Greater than 3 years	0
Strongly Agree	6.75	Greater than 3 years	1
Strongly Agree	7	Greater than 3 years	0
Strongly Agree	7	Greater than 3 years	0
Strongly Agree	6.92	Greater than 3 years	0
Agree	6	Greater than 3 years	0
Strongly Agree	6.85	Greater than 3 years	0
Strongly Agree	7	Greater than 3 years	2
Strongly Agree	7	Less than three years	0
Strongly Agree	6.62	Greater than 3 years	0
Strongly Agree	7	Less than three years	0
Strongly Agree	6.92	Greater than 3 years	0
Strongly Agree	7	Greater than 3 years	0
Strongly Agree	6.38	Greater than 3 years	0
Strongly Agree	6.62	Less than three years	0
Strongly Agree	6.69	Less than three years	0
Strongly Agree	7	Less than three years	1
Strongly Agree	6.69	Less than three years	0
Strongly Agree	6.85	Greater than 3 years	0
Strongly Agree	6.38	Greater than 3 years	0
Strongly Agree	6.46	Greater than 3 years	0
Strongly Agree	7	Less than three years	0
Strongly Agree	6.85	Greater than 3 years	0
Strongly Agree	6.69	Greater than 3 years	0
Strongly Agree	7	Greater than 3 years	0
Strongly Agree	7	Greater than 3 years	0
Strongly Agree	6.83	Less than three years	1

Strongly Agree	6.62	Greater than 3 years	0
Strongly Agree	7	Greater than 3 years	0
Strongly Disagree	1	Less than three years	0
Strongly Agree	7	Greater than 3 years	0
Strongly Agree	6.69	Greater than 3 years	0
Strongly Agree	7	Less than three years	0
Strongly Agree	6.62	Greater than 3 years	0
Strongly Agree	6.38	Less than three years	0

q10_accessible_1	q10_listener_2	q10_expertise_3	q10_independence_4	q10_critiques_5
3	3	3	3	2
2	2	3	3	2
3	3	3	3	2
3	3	3	3	3
2	3	3	3	3
3	3	3	3	3
3	3	3	3	3
3	3	3	3	3
3	3	3	3	.
3	3	3	3	3
3	3	3	3	3
3	3	3	3	3
2	3	3	3	3
0	2	3	3	2
3	3	3	3	3
3	3	3	3	3
3	3	3	3	3
2	3	3	3	3
3	3	3	3	3
3	3	3	3	2
3	3	3	3	3
3	3	3	3	3
3	3	3	2	2
3	3	3	3	3
3	3	3	3	3
3	2	3	3	3
3	3	3	3	3
3	3	3	2	2
3	3	3	3	3
2	2	2	2	2
3	2	3	2	0
2	3	3	3	-1

2	0	3	2	2
3	2	3	3	3
3	3	3	3	3
3	3	3	3	3
2	2	3	3	1
3	3	3	3	3
3	3	3	3	3
3	3	3	3	3
3	3	3	3	3
3	3	3	3	3
3	3	3	3	3
2	2	2	2	2
3	3	3	3	3
3	3	3	3	3
2	2	3	3	3
3	3	3	3	3
3	3	3	3	3
3	3	3	3	3
3	3	3	3	3
3	3	3	3	3
1	3	3	3	3
2	3	3	3	3
2	3	2	2	2
3	3	3	3	3
3	3	3	3	3
3	3	3	3	3
0	1	2	1	0
3	2	3	3	3
3	3	3	3	3
3	3	3	3	1
3	3	3	3	3
0	2	3	2	3
2	2	3	2	2
3	3	3	3	3
3	3	3	3	3
3	3	3	3	3
3	3	3	2	2

3	2	3	2	3
2	2	3	2	3
3	2	3	2	3
3	3	3	3	3
3	3	3	.	2
2	2	2	.	2
3	3	3	3	3
3	3	3	3	3
3	3	3	3	.
3	3	3	3	.
3	3	3	3	3
2	2	3	2	.
3	3	3	3	3
3	3	3	3	2
2	2	3	3	3
3	3	3	3	3
3	3	3	3	3
3	2	2	3	2
3	3	3	3	3
3	3	3	3	2
3	2	3	3	2
3	3	3	3	3
3	3	3	3	3
-2	2	2	0	-3
3	3	3	3	3
3	2	3	2	3
2	3	3	3	2
3	2	3	2	2
3	2	3	3	3
3	3	3	3	3
2	2	2	3	2

3	3	3	2	2
2	2	3	3	2
3	3	3	2	1
2	2	2	2	1
3	3	3	3	3
3	3	3	3	3
3	3	3	3	3
1	0	1	3	0
1	2	3	3	1
3	3	3	3	1
3	3	3	3	3
3	3	3	3	3
3	3	3	3	3
3	3	3	2	2
3	3	3	3	2
3	3	3	3	.
3	3	3	3	3
3	3	3	.	3
3	3	3	3	3
3	3	3	3	3
3	3	3	3	3
2	2	2	2	2
3	3	3	3	2
3	3	3	3	.
3	3	3	3	3
3	2	3	3	3
3	3	3	3	3
3	3	3	3	3
3	3	3	3	3
2	3	3	3	3
3	2	3	3	3
3	3	3	3	2
3	3	3	3	3
3	3	3	3	2
3	3	3	3	2
2	3	3	3	2
2	3	3	3	3
3	3	3	3	3
3	3	3	3	3
2	2	3	3	2
3	3	3	3	3
3	3	3	3	3
2	3	3	3	3

3	2	3	2	2
3	3	3	3	3
-3	-3	-3	-3	-3
3	3	3	3	3
3	3	3	3	2
3	3	3	3	3
3	3	3	3	3
3	2	3	2	2

q10_motivates_6	q10_direction_7	q10_ackowledges_8	q10_interest_9	q10_goals_10
3	3	3	3	2
2	2	3	3	2
2	3	3	3	2
3	3	3	3	3
3	3	3	3	2
3	3	3	3	2
3	3	3	3	3
3	3	3	3	3
3	3	.	2	2
3	3	3	3	3
3	3	3	3	3
3	3	3	3	3
2	3	3	3	3
3	3	3	3	2
.	3	.	3	3
3	3	3	3	3
3	3	3	3	3
3	2	3	3	2
2	3	3	3	2
2	3	2	2	2
3	3	3	3	3
3	3	3	3	3
2	3	2	3	2
3	3	3	3	3
3	3	3	3	2
3	3	3	3	3
3	3	3	3	3
3	2	2	3	2
3	3	3	3	3
2	2	2	2	2
1	2	3	3	2
1	2	3	3	0

2	2	2	3	2
2	3	3	3	2
3	3	3	3	3
3	3	3	3	3
1	3	2	3	3
3	3	3	3	3
3	3	3	3	3
3	3	3	3	3
3	3	3	3	2
3	3	3	3	3
3	3	3	3	3
2	2	2	2	2
3	3	3	3	2
3	3	3	3	3
3	2	3	3	3
3	2	3	3	3
3	3	3	3	3
3	3	3	3	3
3	3	3	3	3
3	3	3	3	3
3	2	2	2	3
2	3	3	3	3
2	2	2	3	2
3	3	3	3	3
3	3	3	3	1
3	3	3	3	3
1	0	1	0	2
2	2	3	3	2
3	3	3	3	3
2	3	2	3	3
3	3	3	3	3
2	1	3	3	2
3	3	3	2	2
3	3	3	3	3
3	3	3	3	3
3	3	3	3	3
2	2	3	1	2

3	3	2	2	2
3	2	3	2	2
3	2	2	2	3
3	3	3	3	3
2	1	2	1	2
2	2	2	2	.
3	3	3	3	3
3	3	3	3	3
.	2	.	2	2
3	3	3	3	3
3	3	3	3	3
.	2	.	2	2
3	3	3	3	2
3	3	3	3	3
1	3	3	3	1
3	2	3	3	3
3	3	3	3	3
1	2	3	3	3
3	3	2	3	3
2	3	3	3	3
2	1	2	3	1
3	3	3	3	3
3	3	3	3	3
-3	0	2	-2	0
3	3	3	3	3
3	3	3	3	2
2	2	3	3	3
2	2	2	3	3
3	2	3	3	1
3	3	3	3	3
2	2	2	3	2

2	3	2	2	2
3	3	3	3	3
2	2	3	3	2
1	2	2	2	1
3	3	2	3	3
3	3	3	3	3
3	3	3	3	3
0	0	0	1	0
2	1	3	3	2
3	3	2	3	3
3	3	3	3	3
3	3	3	3	3
3	3	3	3	3
2	3	3	3	3
2	3	3	3	3
2	3	3	3	3
3	3	3	3	3
2	3	3	3	2
3	3	3	3	3
3	3	3	3	3
2	3	3	3	3
2	2	2	2	2
2	3	3	3	3
3	3	.	3	3
3	3	3	3	3
3	3	3	3	2
3	3	3	3	3
3	3	3	3	3
3	3	3	3	3
3	1	2	3	2
2	2	3	3	3
2	3	3	3	2
3	3	3	3	3
3	2	3	3	3
2	3	3	3	3
1	2	3	3	2
3	3	3	3	2
3	3	3	3	3
3	3	3	3	2
3	3	3	3	2
3	3	3	3	3
3	3	3	3	3
3	.	3	3	3

3	3	3	3	2
3	3	3	3	3
-3	-3	-3	-3	-3
3	3	3	3	3
2	3	3	3	3
3	3	3	3	3
2	2	3	3	1
3	2	3	3	2

q10_network_11	q10_advice_12	q10_balance_13	q10_overall	Mean Mentor Score (-3,-2,-1,0,1,2,3)
3	3	2	3	2.77
3	2	3	3	2.46
3	2	2	3	2.62
3	3	3	3	3.00
3	3	3	3	2.85
3	3	3	3	2.92
3	3	3	3	3.00
3	3	3	3	3.00
2	.	3	3	2.70
3	3	.	3	3.00
2	3	2	3	2.85
3	3	3	3	3.00
3	3	3	3	2.85
2	3	3	3	2.46
.	3	.	3	3.00
3	3	3	3	3.00
3	3	3	3	3.00
2	3	3	3	2.69
2	2	3	3	2.69
1	2	2	2	2.31
3	3	3	3	3.00
3	3	3	3	3.00
2	3	3	3	2.54
3	3	3	3	3.00
3	3	2	3	2.85
3	3	1	3	2.77
3	3	1	3	2.85
2	3	2	3	2.46
3	3	3	3	3.00
2	1	3	3	2.00
2	0	3	2	2.00
2	-1	2	2	1.69

2	2	0	2	1.85
3	3	3	3	2.77
3	.	.	3	3.00
3	3	3	3	3.00
3	2	3	3	2.38
3	3	3	3	3.00
3	3	3	3	3.00
3	3	3	3	3.00
2	3	3	3	2.85
1	3	3	3	2.85
3	3	3	3	3.00
2	2	2	2	2.00
2	2	3	3	2.77
2	3	2	3	2.85
3	3	3	3	2.77
3	3	2	3	2.85
3	3	3	3	3.00
3	3	3	3	3.00
3	3	3	3	3.00
3	3	3	3	3.00
2	3	3	3	2.54
2	.	2	3	2.67
2	2	2	2	2.15
3	3	3	3	3.00
1	3	3	3	2.69
3	3	3	3	3.00
-1	0	0	1	0.54
3	3	3	3	2.69
3	3	3	3	3.00
3	3	2	3	2.62
3	3	3	3	3.00
2	2	3	2	2.15
2	2	2	3	2.31
3	3	3	3	3.00
3	3	3	3	3.00
3	3	3	3	3.00
0	3	0	3	2.00

2	2	2	2	2.38
2	3	3	3	2.46
.	3	.	2	2.55
3	3	3	3	3.00
2	3	.	3	2.18
.	2	2	2	2.00
3	3	3	3	3.00
.	3	3	3	3.00
2	2	2	2	2.40
.	.	3	3	3.00
3	3	3	3	3.00
2	.	.	3	2.13
1	2	3	3	2.69
2	3	3	3	2.85
3	2	3	1	2.46
1	3	2	3	2.69
3	3	3	3	3.00
3	1	3	3	2.38
2	2	2	3	2.69
3	3	3	3	2.85
0	2	2	2	2.00
3	3	3	3	3.00
3	3	3	3	3.00
-2	-3	.	-2	-0.75
3	3	3	3	3.00
3	3	2	3	2.69
3	2	.	3	2.58
3	3	3	3	2.54
2	3	2	3	2.54
3	3	3	3	3.00
2	2	2	2	2.15

3	2	3	3	2.46
3	3	2	3	2.69
2	1	3	2	2.31
2	.	2	1	1.75
3	3	3	3	2.92
3	3	3	3	3.00
2	2	3	3	2.85
0	0	2	0	0.62
3	2	3	3	2.23
1	3	3	3	2.62
3	3	3	3	3.00
3	3	3	3	3.00
3	3	3	3	3.00
2	3	3	3	2.69
3	3	3	3	2.85
3	.	3	3	2.91
3	3	3	3	3.00
2	3	3	3	2.75
3	3	3	3	3.00
3	3	3	3	3.00
3	3	3	3	2.92
2	2	2	2	2.00
3	3	3	3	2.85
3	3	3	3	3.00
3	3	3	3	3.00
2	3	1	3	2.62
3	3	3	3	3.00
3	3	2	3	2.92
3	3	3	3	3.00
1	2	3	3	2.38
1	3	3	3	2.62
2	3	3	3	2.69
3	.	3	3	3.00
2	2	3	3	2.69
3	3	3	3	2.85
2	2	3	3	2.38
1	0	3	3	2.46
3	3	3	3	3.00
2	3	3	3	2.85
3	3	3	3	2.69
3	3	3	3	3.00
3	3	3	3	3.00
2	3	3	3	2.83

3	3	2	3	2.62
3	3	3	3	3.00
-3	-3	-3	-3	-3.00
3	3	3	3	3.00
2	2	3	3	2.69
3	3	3	3	3.00
3	2	3	3	2.62
2	2	2	3	2.38

Mean Mentor Score (1,2,3,4,5,6,7)
6.77
6.46
6.62
7.00
6.85
6.92
7.00
7.00
6.70
7.00
6.85
7.00
6.85
6.46
7.00
7.00
7.00
6.69
6.69
6.31
7.00
7.00
6.54
7.00
6.85
6.77
6.85
6.46
7.00
6.00
6.00
5.69

5.85
6.77
7.00
7.00
6.38
7.00
7.00
7.00
6.85
6.85
7.00
6.00
6.77
6.85
6.77
6.85
7.00
7.00
7.00
7.00
6.54
6.67
6.15
7.00
6.69
7.00
4.54
6.69
7.00
6.62
7.00
6.15
6.31
7.00
7.00
7.00
6.00

6.38
6.46
6.55
7.00
6.18
6.00
7.00
7.00
6.40
7.00
7.00
6.13
6.69
6.85
6.46
6.69
7.00
6.38
6.69
6.85
6.00
7.00
7.00
3.25
7.00
6.69
6.58
6.54
6.54
7.00
6.15

6.46
6.69
6.31
5.75
6.92
7.00
6.85
4.62
6.23
6.62
7.00
7.00
7.00
6.69
6.85
6.91
7.00
6.75
7.00
7.00
6.92
6.00
6.85
7.00
7.00
6.62
7.00
6.92
7.00
6.38
6.62
6.69
7.00
6.69
6.85
6.38
6.46
7.00
6.85
6.69
7.00
7.00
6.83

6.62
7.00
1.00
7.00
6.69
7.00
6.62
6.38
