# Supplementary material for: A new Mentor Evaluation Tool: Evidence of validity
Source: PLoS One. 2020 Jun 16;15(6):e0234345. doi: 10.1371/journal.pone.0234345 (PMC7297334; doi:10.1371/journal.pone.0234345)
Supplement: Attachment — Submitted filename: Response to Reviewers.docx [file pone.0234345.s003.docx]

Dear Editor and Reviewers;

We appreciate your thorough review of our manuscript, “A New Mentor Evaluation Tool: Evidence of Validity” PONE-D-19-35869, and for your insightful suggestions and feedback. Please find our responses to your comments below. The new line numbers indicate where we made the changes according to your suggestion on the marked up version of the manuscript.

Delete period. *Extra period has been deleted. Line 41*

Delete one space. *The space has been deleted. Thank you. Line 94*

In this section i.e. the before lines 105-115 you mentioned tools which have limited utility (only relevant for specific types of mentors or a specific population or not rigorously tested for validity). Regarding the example in these lines it seems not clear what you criticize. Is it that it is only focusing on C&T science mentees? Should be clearer.
*Thank you for your comment. A new sentence was added to clarify that the investigators focused on C&T clinician and scientist mentors’ evaluations. Line 104-106*

As the last sentence of this section is related to what was written before I would add “therefore” => “….such as the MET therefore is essential ….”
*The last sentence of this section was revised as you suggested. Line 152*

How many participants and how many of each group? Specify what you mean with “groups”. The reader probably does not have them in mind anymore, so maybe list them in parenthesis?

*Thank you for your suggestion. We have included the number of mentors in each group and clarified the three groups. We contacted all 30 mentors who have received the Lifetime Achievement in Mentorship Award, all 76 mentors who have completed the Mentor Development Program and 100 mentors who have not received the Lifetime Achievement in Mentorship Award or completed the Mentor Development Program. Line 199-207 and 219-221*

Is the 2014-study the present study you describe in the abstract and under “Design” (Material and Methods)? – this is somehow confusing throughout the manuscript and should be clarified from the beginning (which is/are pilot studies and which is the present study?). In this respect your manuscript is not easy to read and clarification would be a help for the reader.
*We revised the Material and Methods section to clarify which studies were used in the development of the Mentor Evaluation Tool and which part is the current validity study. Line 167-170 and 191-193*

Remove double space to be consistent within your manuscript.
*Double space was removed between the previous line 191 and 192. Line 189-190*

Why did you chose this method that faculty mentors could choose the mentees to be contacted? Why not randomly chose the mentees out of the whole group of each mentor. I think this needs explanation as it could be a bias, i.e. mentor could choose the mentees he/she anticipates most to rate positively. (Later in the discussion you refer to this point as a limitation).
*Thank you for the suggestion. Ideally we would have used this approach, but the UCSF School of Medicine does not have a comprehensive database of mentor / mentee pairs for which to draw from so we had to rely on the mentors to provide the names of their mentees. The study design insured that mentors would not know which mentees evaluated them so as to limit any possible bias. Line 229-231*

Why did you choose 3-5 Mentees? What are your thoughts/ calculations about participant number.
*The rationale for choosing 3-5 mentees per mentors was to include as many diverse mentors as possible. New faculty mentors may only have 3 mentees while experienced mentors will have more than 5 mentees. We wanted to include a wide range of mentors, those who have been a mentor for several years, and those with less experience being a mentor. Line 225-227*

Did the mentees provide their consent that their names and email-addresses were provided?
*Mentees consented before they started the evaluation of their mentors. This will be included in the method section. Line 229-231*

Was this done anonymously? You answer this question only in the discussion.

*Mentees consented anonymously. We will include this in the Material and Method section as well as in the discussion section. Line 229*

Primary contact author:

Michi Yukawa, MD, MPH

Michi.Yukawa@ucsf.edu
