# Supplementary material for: A new Mentor Evaluation Tool: Evidence of validity
Source: PLoS One. 2020 Jun 16;15(6):e0234345. doi: 10.1371/journal.pone.0234345 (PMC7297334; doi:10.1371/journal.pone.0234345)
Supplement: Attachment — Submitted filename: A New Mentor Evaluation Tool- Evidence of Validity.docx [file pone.0234345.s004.docx]

PLOS One: A New Mentor Evaluation Tool: Evidence of Validity

Thank you for the work on the mentoring paper and the revisions. You touch on a critical topic – that of evaluating whether the mentorship that is being provided is actually effective. Please take these comments with their intended purpose to enhance, not critique, your work.

Your introduction would be further strengthened by underscoring the negative consequences of poor mentors. That is the catalyst of creating an evaluation tool which measures the effectiveness of a mentoring relationship. It could inform further mentor training.

L42 – Numbers under 10 are usually spelled out (this is throughout the document).

L127-131 – The definition of mentorship described here is outdated. It is no longer an older, wiser, more experienced singular individual within the same industry. The more contemporary approach is a team of diverse mentors which may include someone senior to you but also those junior to you and at your level. If you are using the binary hierarchical definition of mentorship you should recognize that this is a limitation and it should be stated as such.

For maximum impact, the research done here needs to be triangulated. Surveys are great but they miss answering the “why” question. That’s where interviews and focus groups could fill in the gap and would either affirm or disaffirm the survey data. This might also differentiate between the three groups.

L356 – You mentioned the survey was sent out three times over two months. You need to list the two month duration in your methods section as well.
