# Supplementary material for: A new Mentor Evaluation Tool: Evidence of validity
Source: PLoS One. 2020 Jun 16;15(6):e0234345. doi: 10.1371/journal.pone.0234345 (PMC7297334; doi:10.1371/journal.pone.0234345)
Supplement: Attachment — Submitted filename: Response to reviewers_5_2020.docx [file pone.0234345.s005.docx]

Dear Editors and Reviewers;	May 9, 2020

Thank you very much for your thoughtful comments and review of our manuscript “A New Mentor Evaluation Tool: Evidence of Validity” PONE-D-19-35869R1. We have responded to all of your suggestions below. The new line numbers correspond to the manuscript with track changes.

Reviewer #1: The author addressed all my previous comments satisfactorily. I therefore recommend publication of this manuscript on the condition that 3 grammatical errors are corrected:

Line 170: change "the relevant literatures were reviewed" to "the relevant literature was reviewed"

*Thank you very much for your suggestion. The sentence was changed. (Line 188)*

Line 196: change "rated more higly" to "rated higher"

*Thank you very much for your edit. The sentence was changed. (Line 214)*

Line 227: change "greater than five mentees" to "more than five mentees"

*Thank you very much for your suggestion. The sentence was changed. (Line 247)*

Reviewer #2:

Your introduction would be further strengthened by underscoring the negative consequences of poor mentors. That is the catalyst of creating an evaluation tool, which measures the effectiveness of a mentoring relationship. It could inform further mentor training.

*We appreciate your suggestion. We added the following sentence to the Introduction: “Conversely, previous research has shown that failed mentorship may contribute to mentees not obtaining grant funding and leaving academic careers, among other negative outcomes (7,8).” (Lines 59-64)*

L42 – Numbers under 10 are usually spelled out (this is throughout the document).

*Thank you very much for your correction. All numbers under 10 now are spelled out throughout the manuscript.*

L127-131 – The definition of mentorship described here is outdated. It is no longer an older, wiser, more experienced singular individual within the same industry. The more contemporary approach is a team of diverse mentors which may include someone senior to you but also those junior to you and at your level. If you are using the binary hierarchical definition of mentorship you should recognize that this is a limitation and it should be stated as such.

*Thank you very much for your comment. We agree that team mentoring, peer mentoring etc. are very important to mentee success. We have added the following verbiage to better incorporate your suggestion, including a more recent definition of mentorship: The National Research Mentoring Network defines mentoring as: “A mutually beneficial, collaborative learning relationship that has the primary goal of helping mentees acquire the essential competencies needed for success in their chosen career. It includes using one’s own experience to guide another person through an experience that requires personal and intellectual growth and development” (28). We acknowledge that other mentoring models that incorporate team mentoring, peer mentoring, and distant and web-based mentoring are also important to mentee success.(7) As a dyadic mentoring relationship is often a key component of many mentoring programs, we chose to focus on this context of mentorship as we developed our tool. (Lines 138-149).*

*Also, as you suggest, we have added this issue to the discussion of study limitations. (Line 405-408)*

For maximum impact, the research done here needs to be triangulated. Surveys are great but they miss answering the “why” question. That’s where interviews and focus groups could fill in the gap and would either affirm or disaffirm the survey data. This might also differentiate between the three groups.

*Thank you very much for your suggestion. We agree that focus groups conducted among the mentees of three different groups of mentors would have provided more insightful data and may have differentiated among the three groups. We have added this as another one of our study limitations and something to explore in the future. (Line 408-411)*

L356 – You mentioned the survey was sent out three times over two months. You need to list the two month duration in your methods section as well.

*Thank you very much for your correction. We have added this to the methods section. (Line 227-229 and 402)*
